# Research progress on deep learning in magnetic resonance imaging–based diagnosis and treatment of prostate cancer: a review on the current status and perspectives

**DOI:** 10.3389/fonc.2023.1189370

**Published:** 2023-06-13

**Authors:** Mingze He, Yu Cao, Changliang Chi, Xinyi Yang, Rzayev Ramin, Shuowen Wang, Guodong Yang, Otabek Mukhtorov, Liqun Zhang, Anton Kazantsev, Mikhail Enikeev, Kebang Hu

**Affiliations:** ^1^ Institute for Urology and Reproductive Health, I.M. Sechenov First Moscow State Medical University (Sechenov University), Moscow, Russia; ^2^ I.M. Sechenov First Moscow State Medical University (Sechenov University), Moscow, Russia; ^3^ Department of Urology, The First Hospital of Jilin University (Lequn Branch), Changchun, Jilin, China; ^4^ Department of Radiology, The Second University Clinic, I.M. Sechenov First Moscow State Medical University (Sechenov University), Moscow, Russia; ^5^ Regional State Budgetary Health Care Institution, Kostroma Regional Clinical Hospital named after Korolev E.I. Avenue Mira, Kostroma, Russia; ^6^ School of Biomedical Engineering, Faculty of Medicine, Dalian University of Technology, Dalian, Liaoning, China

**Keywords:** deep learning, machine learning, computer-aided diagnosis, prostate cancer, radiotherapy, precision therapy

## Abstract

Multiparametric magnetic resonance imaging (mpMRI) has emerged as a first-line screening and diagnostic tool for prostate cancer, aiding in treatment selection and noninvasive radiotherapy guidance. However, the manual interpretation of MRI data is challenging and time-consuming, which may impact sensitivity and specificity. With recent technological advances, artificial intelligence (AI) in the form of computer-aided diagnosis (CAD) based on MRI data has been applied to prostate cancer diagnosis and treatment. Among AI techniques, deep learning involving convolutional neural networks contributes to detection, segmentation, scoring, grading, and prognostic evaluation of prostate cancer. CAD systems have automatic operation, rapid processing, and accuracy, incorporating multiple sequences of multiparametric MRI data of the prostate gland into the deep learning model. Thus, they have become a research direction of great interest, especially in smart healthcare. This review highlights the current progress of deep learning technology in MRI-based diagnosis and treatment of prostate cancer. The key elements of deep learning-based MRI image processing in CAD systems and radiotherapy of prostate cancer are briefly described, making it understandable not only for radiologists but also for general physicians without specialized imaging interpretation training. Deep learning technology enables lesion identification, detection, and segmentation, grading and scoring of prostate cancer, and prediction of postoperative recurrence and prognostic outcomes. The diagnostic accuracy of deep learning can be improved by optimizing models and algorithms, expanding medical database resources, and combining multi-omics data and comprehensive analysis of various morphological data. Deep learning has the potential to become the key diagnostic method in prostate cancer diagnosis and treatment in the future.

## 1 Introduction

Prostate cancer (PCa) is a commonly occurring urological malignancy among middle-aged and older men, with its incidence on the rise. In 2020, there were approximately 1.4 million new PCa cases reported globally, resulting in around 375,000 deaths, making it the second most common cancer among men, following lung cancer, and the fifth leading cause of cancer-related deaths among men (1). Although digital rectal examination and prostate-specific antigen (PSA) test are routinely conducted for the diagnosis of PCa, transrectal ultrasound (TRUS) has been the primary imaging technique for clinical suspicion and diagnosis of PCa in the past (2, 3). However, due to its low sensitivity and specificity, particularly for detecting lesions present in the transitional zone (TZ), mpMRI has replaced TRUS as the first-line radiological screening modality for clinical suspicion of PCa (4–6). Compared with other imaging examinations, MRI of the prostate provides a higher soft-tissue resolution and multiple imaging data parameters non-invasively, which facilitates better understanding of the complete prostate gland and its relationship with the surrounding environment and also provides improved guidance for PCa staging (7, 8). Therefore, MRI has become the preferable imaging tool for patients with suspected PCa or those at risk of PCa (9–11). The prostate imaging-reporting and data system (PI-RADS) provides a comprehensive set of standards for scanning, interpreting, and reporting mpMRI (12). Combining mpMRI with PI-RADS scaling results in more precise PCa diagnosis and staging, as well as improved guidance for later biopsies, and has contributed significantly to reducing overdiagnosis (13–15). Although mpMRI is a valuable technique in PCa diagnosis, manual interpretation of mpMRI data is complex, time-consuming, and challenging due to low sensitivity and specificity of the interpreting results (16–18). Deep learning (DL) technology has the capability to mine various features from medical images that are difficult to identify and distinguish using the naked eye in the macroscopic view (19, 20). DL technology can guide clinicians in medical diagnosis and help reduce diagnostic accuracy issues caused by factors mentioned above, providing physicians with accurate disease information (21, 22). Over the past few years, several computer-aided diagnosis (CAD) systems have been applied to PCa diagnosis with positive outcomes (23–26). CAD systems can be classified into two categories: computer-aided detection (CADe) and computer-aided diagnosis (CADx) (27, 28). CADe can determine if a patient has PCa and localize the possible PCa lesion based on the entire mpMRI data. CADx can evaluate a series of manually or automatically selected tumor-suspected areas by radiologists or CADe systems, followed by assessing and evaluating the aggressiveness of PCa (24, 29–31). This review presents a cross-disciplinary summary of research progress in PCa using DL-based CADs to make the artificial intelligence (AI) process understandable to not only radiologists but also general physicians who lack systematic and specialized imaging interpretation training. We briefly describe the application of DL techniques based on prostate MRI data and provide possible research ideas for future studies.

## 2 Overview of deep learning techniques

**Figure 1:**
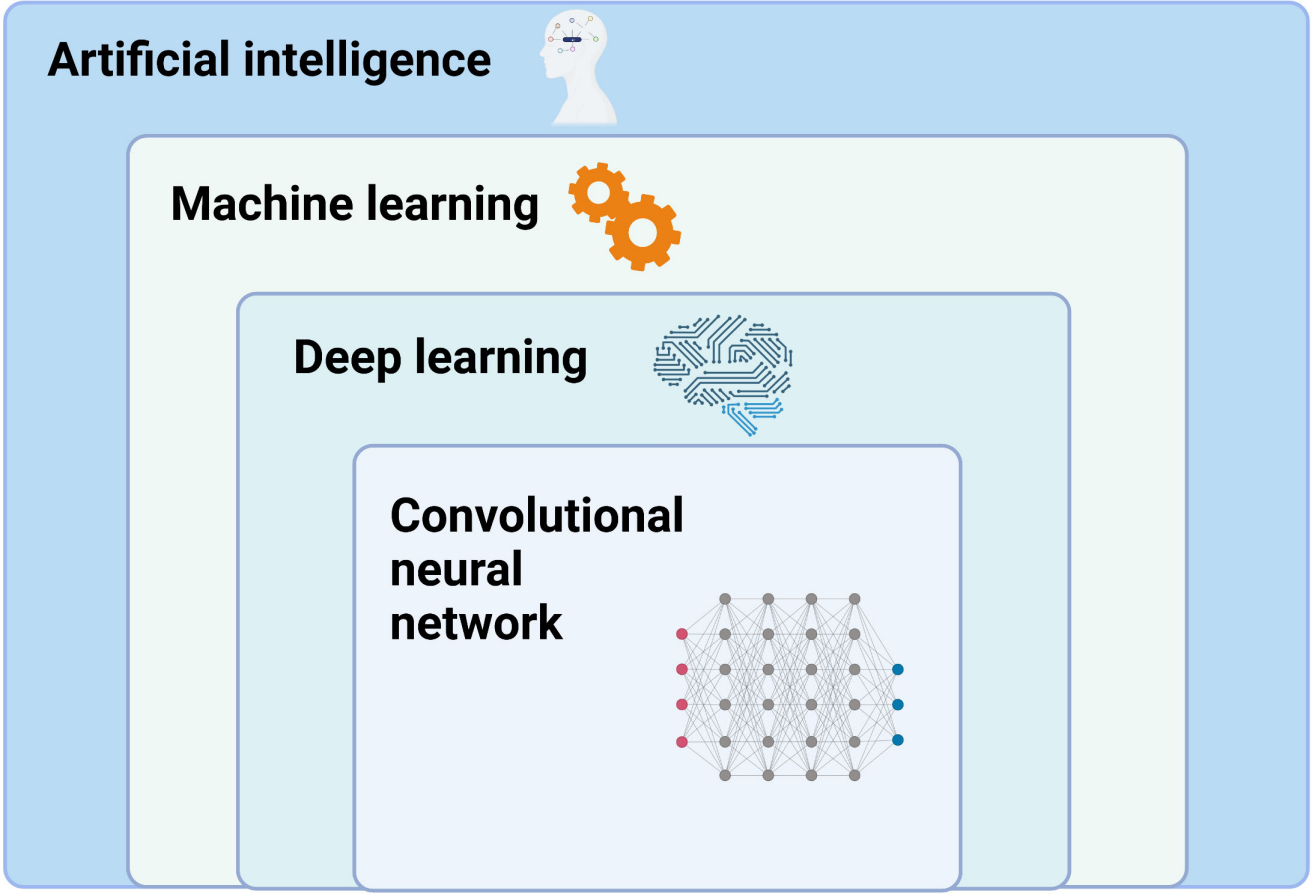
Stratification of artificial intelligence. Created with BioRender.com.

According to the latest Prostate Imaging-Reporting and Data System Version 2.1 (PI-RADS 2.1) recommendations, mpMRI image sequences for PCa detection and diagnosis typically consist of T_2_-weighted imaging (T_2_W), diffusion-weighted imaging (DWI), average diffusion coefficient (ADC) maps, and dynamic contrast-enhanced (DCE) imaging (12). DWI and ADC sequences are primarily employed to detect peripheral zone lesions, while T_2_W focuses on detecting transition zone lesions (32, 33). The PI-RADS score, calculated from mpMRI data, ranges from 1 (low likelihood of clinically significant PCa) to 5 (high likelihood of clinically significant PCa) and serves as a crucial diagnostic measure to determine the necessity of a biopsy (34, 35). Due to its significant clinical value, PI-RADS recommendations have been updated for standardizing prostate MRI scanning and interpretation processes. However, accurately interpreting mpMRI data requires a high level of expertise and skill. Furthermore, inter-observer and intra-observer variability values, which pertain to different radiologists interpreting the same MRI results and a single radiologist interpreting the same MRI results multiple times, respectively, tend to exhibit high variability (36, 37). This affects the broader utilization of mpMRI in PCa diagnosis. Consequently, to reduce interpretation time, enhance image interpretation quality, and minimize the risk of overtreatment, DL has emerged as the predominant AI method within machine learning (ML) technology (38, 39). Inspired by human learning patterns, ML can be broadly categorized into supervised learning, which utilizes well-labeled training data examples with fully controlled data input and output, and unsupervised learning, which operates on unlabeled datasets and aims to identify correlations within the dataset (38–40). Semi-supervised learning, a hybrid mode between supervised and unsupervised learning, uses partially labeled training data while the remainder stays unlabeled (39, 41, 42). Another renowned learning framework, reinforcement learning, obtains feedback based on each action’s response in the environment, modifying model parameters to maximize anticipated benefits (39, 43). DL technology, introduced by Hinton in 2006 (44), is an ML subset sharing similar working principles but featuring an advanced multilayer neural network that mimics human biological neural networks for data representation learning (39, 45). A key distinction between ML and DL lies in feature extraction methods, where conventional ML relies on hand-crafted approaches by expert specialists, and DL automatically extracts features within network layers (45, 46). As hand-crafted feature design demands significant effort and considerable workload, a growing number of DL algorithm (DLA) are emerging, tending to replace ML in medical image processing (20, 45, 47). However, hand-crafted approaches persist in situations with limited annotated data, and several studies have demonstrated promising results by fusing models with classic hand-crafted features and DL-extracted features (46, 48, 49). Typically, the majority of CAD systems for PCa involve processing steps such as imaging alignment, prostate localization and segmentation, feature-based lesion detection, and task-based classification (16, 24). With the increasing volume of labeled imaging data, supervised learning models represented by convolutional neural networks (CNNs) and non-supervised learning frameworks, including generative adversarial networks (GANs), have been incorporated into various medical imaging processes (50–53). In general, DLA-based analysis constitutes a new form of computer-aided diagnosis that facilitates the automatic acquisition of data-related features and identification of lesions without the requirement of manual segmentation, provided that the dataset is sufficiently large. This approach is innovative in that it allows for spontaneous learning of features, resulting in improved efficiency and accuracy of lesion detection (16, 20, 45, 54) (**Figure 1**).

### 2.1 Main working principles of DLA (**Figure 2A**)

**Figure 2:**
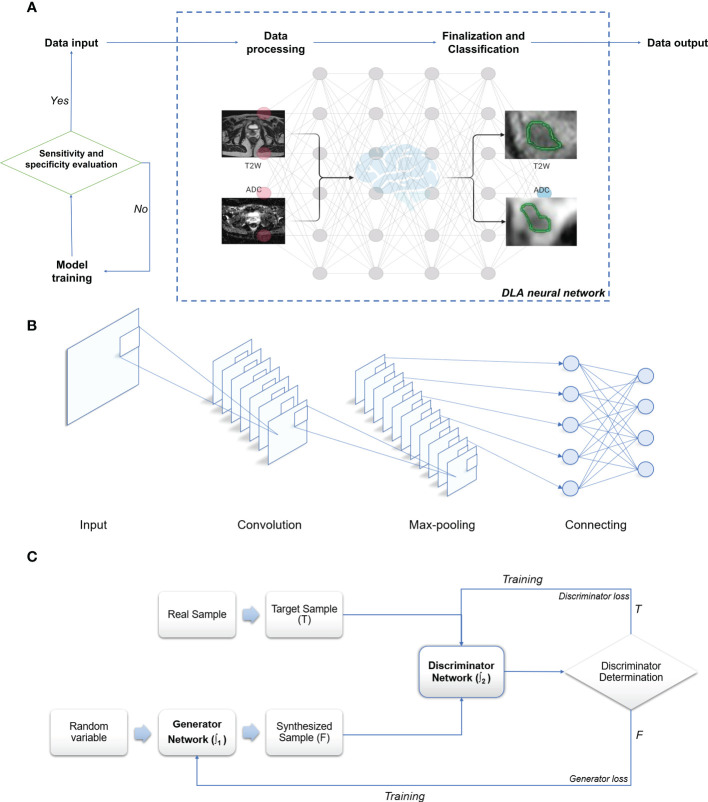
Schematic illustration of deep learning algorithm. **(A)** Working algorithm of image processing. **(B)** Principle and architecture of CNN. **(C)** Principle and architecture of GAN: T, True data; F, False data; ∫1 **-** Function 1 of the generator network; ∫2 **-** Function 2 of the discriminator network. Created with BioRender.com.

In summary, within the medical imaging domain, DLAs perform tasks such as image classification, object detection, and semantic segmentation. Image classification determines benignity or malignancy, tumor types, grading, and staging from medical images; object detection localizes tumors and extracts their information from images; and semantic segmentation outlines tumors or adjacent organs in the images (54–57). Among the various DL-based models, deep CNNs have garnered significant attention due to their promising performance in medical imaging (52, 58). The multilayer neural network structure of DLAs, inspired by the human visual system, has the potential to process convolution operations (38, 52, 59). CNN structures primarily consist of a convolutional layer, max-pooling layer, and fully connected layer (20, 38, 58–61). These layers pertain to specific calculation methods or functions that receive, compute, and output relevant data. The convolution layer, the core of CNNs, extracts image features by constructing multiple convolution kernels (60). The max-pooling layer, also known as the down-sampling layer, reduces computational effort by consolidating data within a certain range. The fully connected layer, used as a classifier, integrates all local information acquired from the previous max-pooling or convolutional layer that is class-distinctive, ultimately producing the desired class predictions (58, 61, 62). In brief, the convolutional layer of a CNN functions as a feature extractor, while the fully connected layer serves as a classifier (63) (**Figure 2B**). The trained CNN forms its network structure and weight files, which are the foundation for predicting the same type of unknown data. Function-dependent networks based on CNN have been designed for specific computer vision tasks, such as AlexNet and ResNet for image classification (64, 65), YOLO and Faster R-CNN for object detection (66, 67), and U-Net and Mask R-CNN for semantic segmentation (58, 61, 62, 68). Another valuable deep learning network, the GAN, has demonstrated effectiveness for semi-supervised learning (69), supervised learning (70), and reinforcement learning (71), despite its initial proposal for unsupervised learning. GAN can be simply described as a deep learning model used to create alternative imaging data similar to the target data, but with improved quality and reduced noise (51, 53, 72). GAN primarily consists of two separate but interdependent neural networks, functioning as the generator and the discriminator. Generated data (false) from the generator, using random variables as input, or target data (true) are then input into the discriminator (53, 72). These two networks are trained competitively and adversarially, with the aim of making the discriminator model strictly capable of distinguishing between the synthesized data generated by the generator and the true data, while the generator intends to create data as realistic as possible compared to the target data (53, 72, 73). The primary objective of GAN is to render the discriminator network incapable of differentiating between the output data generated by the generator network and real data (**Figure 2C**). More recently, deep convolutional GANs (DCGANs) have emerged by combining CNNs and GANs to achieve better performance and effectiveness, resulting in their increasing popularity for designing various computer-aided diagnosis (CADx) models (53, 74–76).

### 2.2 Evaluation metrics of DLA

Evaluation metrics for DLA in medical imaging applications encompass accuracy, specificity, sensitivity, dice similarity coefficient (DSC), Jaccard index, receiver operating characteristic (ROC) curve, and area under the ROC curve (AUC) (77–81). While general clinicians need not fully grasp the complex equations and processes involved in these evaluation metrics, a basic understanding of their core principles is crucial for accurately interpreting the performance of pertinent DL models. Sensitivity denotes the likelihood of detecting a positive sample within a positive population (1.2.1), while specificity refers to the probability of identifying a negative result in a negative population (1.2.2) (77). DSC (1.2.3) and the Jaccard index are promising evaluation metrics for assessing segmentation quality. They are typically employed to calculate the similarity between two samples, with values ranging from 0 to 1. A value closer to 1 indicates a better model performance (78, 82). The ROC curve is used to evaluate the diagnostic accuracy and performance of various models. A curve closer to the upper left corner signifies higher diagnostic value, and a larger AUC corresponds to greater application value (79–81). AUC serves as a criterion for determining the quality of classification models, referring to the likelihood of positive examples ranking higher than negative examples in prediction outcomes. An AUC between 0.5 and 1 implies that the model possesses predictive value, and a value closer to 1 signifies superior model performance (79–81).


1.2.1 $$Sensitivity=\frac{TP}{\left(TP+FN\right)}$$

1.2.2 $$Specificity=\frac{TN}{\left(TN+FP\right)}$$

1.2.3 $$\text{DSC}=\frac{2_{*}|X\cap Y|}{\left|X\right|+\left|Y\right|}$$

## 3 Application of mpMRI-based DLA on PCa

### 3.1 Diagnosis of PCa

#### 3.1.1 Detection and classification of PCa

In the clinical management of PCa, accurately distinguishing between low-risk and high-risk cases is crucial to prevent overdiagnosis or delayed treatment (92). For patients with low-risk PCa, mpMRI serves as the primary imaging technique to determine if the lesion has grown or metastasized and to assess disease progression during active surveillance (93). Therefore, a reliable noninvasive assessment system is of significant importance. Fusco et al. (94) performed a systematic literature review, reporting that MRI holds considerable clinical value in localizing and staging PCa. Vente et al. (95) developed a multitasking U-Net model using T_2_W and DWI sequences of MRI, capable of simultaneously detecting and grading PCa with excellent diagnostic outcomes. Wang et al. (96) designed an end-to-end CNN comprising two sub-networks: one for aligning apparent DWI and T_2_W, and the other as a convolutional neural classification network. The end-to-end CNN model was trained and assessed on 360 patients using a fivefold cross-validation method, ultimately exhibiting a sensitivity of 0.89 for identifying high-risk PCa cases. Ishioka et al. (97) developed a fully automated PCa detection system using patients’ T_2_W sequence data, combining two distinct algorithms and demonstrating an AUC of 0.793. Wang et al. (98) compared the detection capabilities of DLAs and non-DLAs in differentiating PCa, using T_2_W sequences from prostate MRI findings of 172 patients, which included 79 patients with PCa and 93 with benign prostatic hyperplasia (BPH). The final ROC curve value was 0.84 for the DL model, compared to 0.70 for the non-DL model. Sanford et al. (99) conducted PI-RADS scoring with a CNN trained on T_2_W/ADC/high-b values, confirming that DLAs possess a PCa assessment potential comparable to clinical PI-RADS scoring. Yang et al. (31) collected T_2_W and DWI sequences from prostate MRI findings of 160 patients and built two parallel deep CNNs. The final features extracted by these two CNNs were input into a classifier based on the support vector machine algorithm, ultimately achieving spontaneous identification of PCa (**Table 1**).

**Table 1:** Currently available models of DL-mpMRI-based PCa detection or segmentation.

Author [Reference]	Year	Sample sizes	MRI sequences	Evaluation
Schelb P. et al. (83)	2019	250 + 62	T2, ADC, DWI	
Xu H. et al. (84)	2019	346	T2, ADC	93.0% Accuracy; 0.95 AUC
Chen Y. et al. (85)	2020	136	T2, ADC	
Arif M. et al. (86)	2020	292	T2, ADC	76.0% Accuracy; 0.89 AUC
Cao R. et al. (17)	2021	427 + 126	T2, ADC	
Ushinsky A. et al. (87)	2021	287	T2	0.898 DSC
Bardis M. et al. (88)	2021	242	T2	0.940 DSC
Soerensen S.J.C. et al. (89)	2021	156	T2	0.92 ± 0.02 DSC
Soni M. et al. (90)	2022	140	T2, ADC	0.654 DSC, 0.695 sensitivity, 0.970 specificity
Li D. et al. (91)	2022	200	T2, DWI, ADC	0.79 AUC

#### 3.1.2 Segmentation of the prostate gland

The clinical measurement of prostate-specific antigen density (PSA-D) is closely related to prostate volume (PV), and PSA-D serves as an indicator of prostate cancer (PCa), with higher PSA-D values suggesting a greater likelihood of clinically significant PCa (100–102). PV is employed to diagnose BPH in clinical settings and assists urologists in selecting suitable surgical procedures and medication strategies for BPH patients (103–105). TRUS is the most common imaging method for calculating PV in clinical practice (106), but it is susceptible to significant measurement errors when the prostate has an irregular shape. Computing PV based on pixel size and layer thickness, in which the prostate gland is segmented on each MRI image, may be more accurate. In a clinical setting, determinization of the type of surgery, such as prostate tissue-preserving surgery and fascial-sparing surgery requires precise differentiation of prostate gland boundaries. Preservation of the neurovascular bundle for performing the nerve-sparing radical prostatectomy (107) to save the erectile function and sparing of the pelvic fascia for fascial-sparing radical prostatectomy (108) to prevent positive surgical margins followed by high risk of clinical recurrences rely on preoperative imaging guidance. In the case of PCa radiotherapy (discussed in more detail later), precise MRI-guided segmentation in radiotherapy significantly improves target accuracy, effectively prevents damage to normal prostate tissues surrounding the tumor, and reduces toxic side effects (109). Hence, accurate, robust, and efficient MRI-guided segmentation of the prostate gland is crucial for evaluating PCa tumors, calculating PV, selecting surgical options for prostate abnormalities, outlining target areas for radiation planning, and monitoring progressive changes in tumor lesions. However, due to heterogeneity in MRI imaging quality and signal intensity, as well as interference from periprostatic tissues and organs like the bladder or rectum, prostate segmentation remains highly challenging (110, 111). Applying DL for accurate prostate gland segmentation on MRI images could facilitate more precise and easy determination of PV and prostate boundaries. CNNs do not require complex feature extraction and are widely utilized for medical image segmentation (112, 113). Zhu et al. (114) developed a three-dimensional (3D) deep learning model containing dense blocks to segment the prostate gland. The 3D structures enable the network to fully exploit the relationship between adjacent images, and the dense blocks make complete use of both shallow and deep information, achieving a DSC of 0.82. Yan et al. (115) proposed a backpropagation neural network that integrates the optimal combination selected from multi-level feature extraction into a single model for prostate MRI image segmentation, achieving a DSC of 0.84, an average increase of 3.19% compared to traditional ML segmentation algorithms based on random forests. To et al. (116) segmented MRI images and identified PCa using a 3D deep dense multipath CNN constructed from T_2_W and ADC sequences, achieving DSCs of 0.95 and 0.89 in two independent test sets, respectively. Dai et al. (117) developed a mask region-based CNN for prostate gland and intraprostatic lesion segmentation, showing that this end-to-end DL model could automatically segment the prostate gland and identify suspicious lesions directly from prostate MRI images without manual intervention, demonstrating its potential to guide clinicians in tumor delineation.

### 3.2 Advanced radiotherapy of PCa

Radiotherapy is a vital component of PCa treatment and relies on a complex series of multimodal medical imaging techniques, such as computed tomography (CT), MRI, cone-beam CT, and positron emission tomography, to localize tumors, establish radiotherapy treatment plans, and assess radiotherapy efficacy (118). Radiotherapy is an indispensable treatment modality for cancer patients, either as neoadjuvant or postoperative therapy, in combination with chemotherapy (119, 120). The main goal of radiotherapy is to maximize the therapeutic gain ratio by delivering an effective radiation dose to the planned target volume (PTV) and avoiding unnecessary radiation exposure to adjacent healthy tissues and organs at risk (OARs) (121, 122). However, manual segmentation of the prostate gland, which is necessary for accurate mapping of PTV and OARs, is prone to errors and can result in less accurate and sensitive outcomes than those desired clinically. In addition, respiratory movements, setup errors, and fluctuations in body weight can lead to displacement of PTV and OARs, potentially resulting in under-measurement of the radiation dose received by the PTV or over-measurement of the radiation dose delivered to OARs (123). To achieve precise tumor localization and appropriate treatment, continuous technological advances have led to the development of precision therapy, such as intensity-modulated radiotherapy (IMRT) and 3D conformal radiation therapy, which aim to provide personalized, precise anticancer treatment by setting an appropriate radiation dose according to the tumor shape while avoiding radiation exposure to OARs as much as possible (124–126). Although precision therapy has improved the accuracy of radiotherapy to some extent, further optimization is necessary to achieve the desired efficacy. Therefore, image-guided adaptive radiotherapy (ART) has emerged as a potential solution to overcome PTV and OAR displacement caused by various factors (123, 127, 128).

#### 3.2.1 ART technology

ART technology allows for systematic monitoring of target lesions and changes in adjacent tissues based on imaging features to optimize radiotherapy plans further (123, 128, 129). ART enables the acquisition of feedback and tracking of target area-related information primarily through offline, online, and real-time modes (129, 130). For instance, offline ART involves measuring setup errors on MRI images obtained during the patient’s initial few treatments, after which the clinical target volume (CTV) coverage is adjusted, and both the dose and treatment plan for subsequent fractions are modified (130, 131). Online ART calculates the necessary data based on the patient’s anatomical imaging information acquired at the time, allowing for modifications to the radiotherapy plan that are directly applied to the current treatment (129–131). Real-time ART involves intra-fraction and inter-beam reprogramming and automatic adjustment of the radiotherapy plan during treatment execution based on dynamic tracking of radiation dose and anatomical details of the target area without manual intervention (129–131). Since the anatomical and geometric variations of PCa are influenced by the degree of bladder and rectal filling, the morphology, location, and volume of PTV and OARs may differ between treatments. Consequently, offline ART is not flexible enough to accommodate these changes (131). While CTV expansion is often used clinically to compensate for these limitations, it can result in increased post-radiotherapy toxicities (132). Although online ART offers improved accuracy compared to offline ART, its time-consuming nature limits its clinical application to some extent. Real-time ART overcomes the drawbacks of both offline and online ART and has been implemented in clinical practice (133), but its safety and robustness require validation due to the lack of a sufficiently comprehensive database for model adaptation and training (134). As mentioned in Section 3.1.2, significant progress has been made in DL-based automatic segmentation of the prostate gland. However, research on developing subsequent radiotherapy systems remains underexplored. Developing an accurate and efficient automated radiotherapy delivery system using DL technology to enhance radiotherapy outcomes has far-reaching clinical implications. Sprouts et al. (135) developed a virtual treatment planner (VTP) based on deep reinforcement learning (DRL) to implement a treatment planning system. The VTP, based on the Q-learning framework, was evaluated using 50 samples, achieving a mean ProKnow plan score of 8.14 ± 1.27 (standard deviation), indicating its potential for IMRT planning in PCa. The application of the conventional ϵ-greedy algorithm for training VTP is time-consuming, restricting its clinical use. Shen et al. (136) introduced a knowledge-guided DRL to adjust treatment plan parameters to enhance VTP training efficiency, achieving a plan quality score of 8.82 ( ± 0.29). Lempart et al. (137) proposed a densely connected DL model based on a modified U-Net, trained on a triplet of 160 patients to predict dose distribution for volumetric-modulated arc therapy. The model maintained the mean percentage error within 1.9% for both CTV and PTV and within 2.6% for OAR, demonstrating its capacity to partially automate the radiotherapy planning process and accelerate treatment progress.

#### 3.2.2 MRI-only radiotherapy

**Figure 3:**
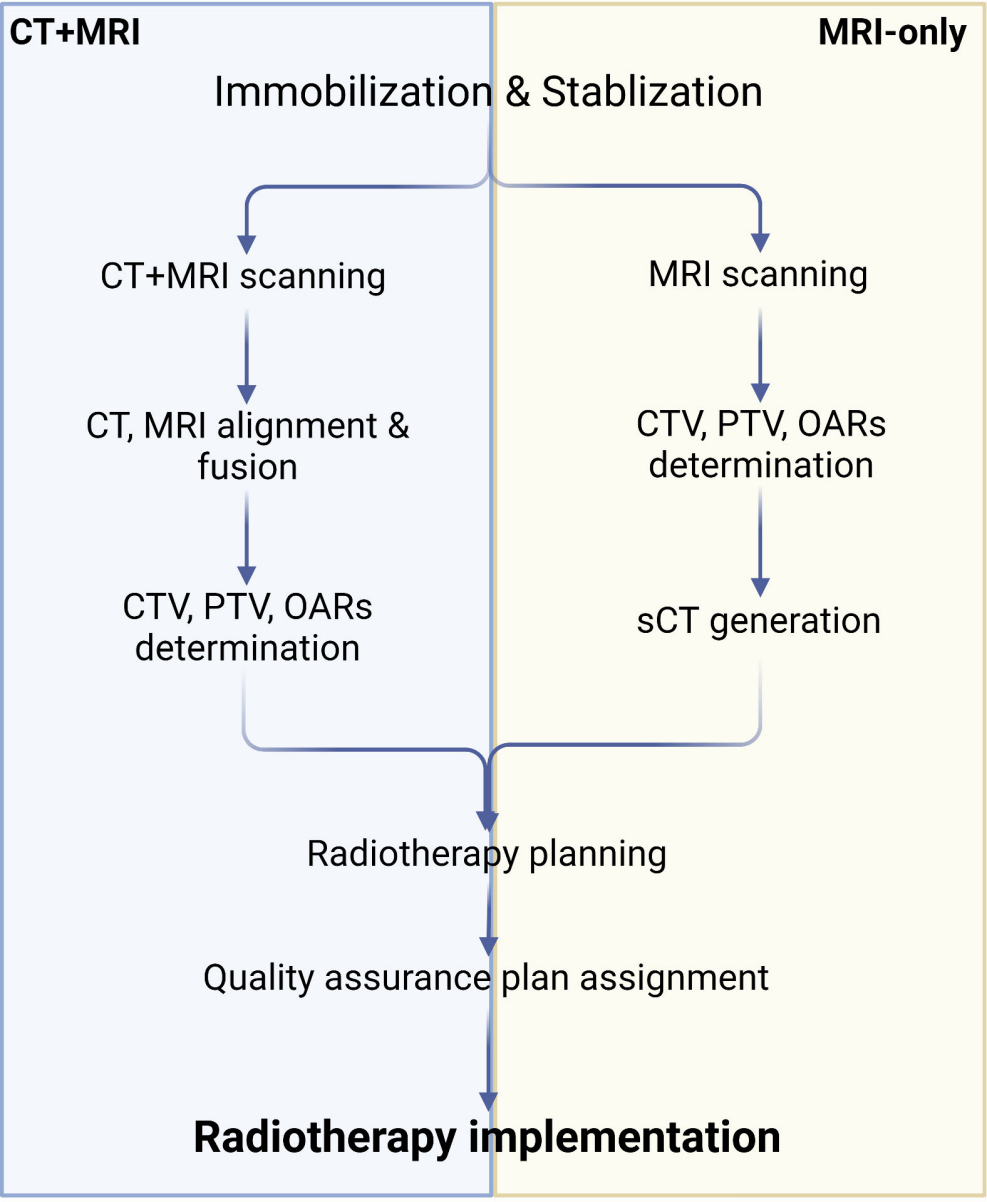
Algorithm of CT+MRI and MRI-only radiotherapy technique. Created with BioRender.com.

Although MRI offers excellent soft-tissue contrast and facilitates relatively precise tumor segmentation, it does not provide the electron density map or Hounsfield units needed for radiation dose calculation. Consequently, it is essential to map relative regions such as CTV, PTV, and OARs on MRI, after which the outlined contours are mapped to CT via image alignment for clinical radiotherapy planning (134, 138, 139). Combining MRI and CT simulations in PCa radiotherapy plans may reduce acute urogenital toxicity (140). However, the labor-intensive process of CT and MRI alignment and the challenges in achieving full alignment can result in systematic errors, potentially leading to dose distribution issues in the target region and diminishing radiotherapy effectiveness (141). To address these problems, recently developed MRI simulators enable the conversion of MRI data to synthetic CT (sCT), allowing radiation dose measurements to directly contribute to radiotherapy planning and establishing MRI-only radiotherapy (138, 139, 142) (**Figure 3**). Common approaches for converting MRI data to sCT include bulk density assignment–based methods, voxel-based methods, and atlas-based methods (143, 144). Currently, a bulk density assignment–based system called magnetic resonance for calculating attenuation (MRCAT™ by Philips) and an atlas-based system called MriPlanner™ (by Spectronic Medical) have been employed for automatic generation of pelvic sCT in clinical practice (145, 146). MriPlanner™ has demonstrated promising performance, as evaluated in the MR-OPERA and MR-PROTECT studies (147, 148). In contrast to sCT generation methods, MRCAT™, due to its bulk density assignment–based nature, requires multiple MRI sequences, such as air, liquid, and bone, each assigned to corresponding electron density or Hounsfield unit values necessary for creating a CT image (144, 149) (**Table 2**). As illustrated previously, the conventional approach to radiotherapy planning, which involves the use of both CT and MRI, necessitates manual intervention for aligning and fusing CT and MRI images, as well as determining the CTV, PTV, and OARs. This manual intervention considerably reduces both accuracy and efficiency. However, by employing DL, which automatically extracts informative features from a large number of training samples to establish a nonlinear mapping from MRI to CT (150), a trained model can swiftly generate highly precise synthetic CT (sCT) images in just a few seconds. These sCT images provide more accurate guidance for ART (151). Fu et al. (152) utilized two-dimensional (2D) and three-dimensional (3D) fully connected CNN based on U-Net to generate pelvic region sCT, with results indicating that accurate sCT was effectively executed using DLA. Conditional GAN, developed by adding a discriminator to U-Net, enable the generated sCT to provide more details, enhancing sCT accuracy and robustness and allowing for more precise radiotherapy planning (153, 154). CycleGAN (cGAN) is a modified adversarial network based on GAN, with additional generators and discriminators incorporated for improved unpaired training data (155, 156). Liu et al. (157) proposed a multi-CycleGAN network and designed a new generator, Z-Net, to improve anatomical details. This approach exhibited lower mean error and mean absolute error and higher dose accuracy of the sCT (**Table 3**).

**Table 2:** MRCAT™ (Magnetic Resonance for Calculating Attenuation) vs. MriPlanner™.

	Magnetic Resonance for Calculating Attenuation	MriPlanner™
Method	Bulk density assignment-based	Atlas-based
Region	Pelvic region	Pelvic region
MRI sequence engaged	Multi-sequences needed (including air, liquid, and bone)	A single sequence is sufficient
Accuracy determination	Determined by the accuracy of segmentation	Determined by the MRI-MRI alignment
Time required	Longer	Less

**Table 3:** Recent designed models of deep learning–based synthetic CT generation in the prostate gland or pelvic region.

Model	Author, Reference	Year	Sample size	Target	Time of sCT generation (s)
U-net	Chen et al. (158)	2018	36	Prostate gland	3.8–7.7
U-net	Arabi et al. (159)	2018	30	Pelvic region	9
cGAN	Nie et al. (160)	2018	21	Pelvic region	30
cGAN	Maspero et al. (161)	2018	32	Pelvic region	5.6
U-net	Fu et al. (152)	2019	16	Prostate gland	5.5
cGAN	Gusumano et al. (162)	2020	40	Pelvic region	175 ± 43

### 3.3 Prognostic assessment of PCa

To improve prognosis monitoring of PCa and reduce mortality, it is essential to consider patients at low risk during active surveillance and those who have undergone radical prostatectomy (163). The European Association of Urology guidelines widely recognize PSA as the primary metric for assessing BCR in clinical practice (164). To mitigate diagnostic bias, leveraging the precise anatomical information provided by MRI is invaluable, as it offers non-invasive insights. This is particularly crucial since PSA levels can fluctuate and be influenced by various factors (165, 166). Furthermore, the role of mpMRI in assessing PCa recurrence has gained importance (167), underscoring the need for comprehensive investigation into MRI-based PCa recurrence prediction. Yan et al. (168) conducted a multicenter study using a DL technique and a novel model called deep radiomic signature for BCR prediction. They combined quantitative features and radiomics extracted from prostate MRI with DL-based survival analysis. The performance of the model was evaluated using data from approximately 600 patients who underwent radical prostatectomy, achieving maximum AUC values of 0.85 and 0.88 for BCR-free survival prediction at 3 years and 5 years, respectively. In addition to recurrence prediction, there should be a significant focus on monitoring metastasis, particularly considering the high occurrence rate of bone metastases in over 80% of patients with advanced PCa (169). The accuracy and sensitivity of conventional bone scintigraphy for detecting skeletal metastases have been questioned (170). Therefore, of the potential in detecting earlier PCa metastasis using PSMA PET-CT and MRI has been established (170, 171). As part of a routine radiological examination for suspected PCa, Liu et al. successfully detected and segmented pelvic bone metastases using dual 3D U-net DLAs rely on T_1_-weighted imaging and diffusion-weighted imaging sequences (172). Through two rounds of evaluation, they achieved a mean DSC value above 0.85 for pelvic bone segmentation and a maximum AUC of 0.85 for metastasis detection, demonstrating accurate detection and segmentation of pelvic bone metastases.

## 4 Discussion

Prostate MRI holds significant potential as a first-line diagnostic and therapeutic approach for prostate gland abnormalities. However, the broader application of prostate MRI is currently limited for various reasons. CNN-based DL models have been employed for fully automatic target segmentation. More importantly, DLA can be easily applied to large-scale samples, making them suitable for real-world clinical practice. In addition to their utility in detecting and segmenting lesions on prostate MRI, DLA have a wide range of applications. Presently, some studies have demonstrated the potential applications of DL in multiple areas. A few studies have employed DL to predict the Gleason score of PCa by using DLAs to assess pathological sections, which demonstrated diagnostic power equal to that of pathologists (173–175). DL has also yielded satisfactory results in prostate gland segmentation on TRUS images (176–178). In radiotherapy, prostate gland and adjacent organ contouring based on DL auto-contouring algorithms may reduce workload and inter-observer variability, as evidenced by several clinical evaluations conducted at different radiotherapy centers (179, 180). Additionally, using DLA for detecting and tracking marker seeds during PCa treatment enhances precise target dosage delivery and minimizes radiation-induced adverse events in normal tissues surrounding the tumor (181, 182). Remarkably, DL has been employed for PI-RADS scoring based on mpMRI of the prostate gland in real-world settings, yielding results similar to PI-RADS scores determined by radiologist experts (83). In recent times, there has been notable progress in integrating DL with nomograms. This integration enables the inclusion of crucial variables such as PSA, PV, patient age, free/total PSA ratio, and PSA-D into the diagnostic process of PCa using MRI data (183, 184).

### 4.1 Limitations and outlook

The research examined in this review highlights the significant potential and wide-ranging prospects of DL applications. Future studies should concentrate on employing DL in prostate MRI for in-depth understanding. Firstly, there is a strong demand for 3D information processing. Presently, most available DLAs rely on 2D images for feature extraction and analysis, indicating that these DLAs may not be suitable for extracting 3D spatial anatomical information from clinically obtained patient images. Although DL has been employed for segmenting 3D medical images of the liver and cardiovascular system (185), there is a scarcity of research on using DL for segmenting 3D images of the prostate gland, necessitating further evaluation. Developing computational segmentation methods appropriate for 3D medical images while preserving the high performance of DL models for PCa detection and diagnosis remains highly challenging. In addition, future research should continually extend to multimodal and multisequence data analyses. Currently, most prostate MRI studies include only T_2_W and DWI sequences. Despite the diminishing role of DCE according to recent PI-RADS guidelines (186), incorporating ADC into the analysis and fusing multiple modalities of feature descriptions for 3D tumor image segmentation may further enhance the accuracy of CNN in identifying PCa. Furthermore, the effectiveness of using DCE sequence in detecting PCa is still debated, given its time-consuming nature and the associated risk of nephrogenic systemic fibrosis (187, 188). Therefore, focusing on biparametric MRI, which assesses only T_2_W and DWI sequences, should be prioritized for rapid screening. Improving CNN architectures may also enhance the computational capabilities of DLAs. Based on cumulative findings, we propose that parallelizing sub-networks analyzing different sequences and then inputting the final result into the classifier or connecting various sub-networks in series and generating the final result directly could yield promising outcomes. Utilizing diverse DLAs, developed by modifying neural network architectures, can further improve detection effectiveness. One primary limitation of DL, not only in medical image processing but also in other professional fields, is the incomprehensibility and lack of interpretability of predictions and decisions made by DLAs (189–191). This becomes critically important in cases where DL-based decisions can result in significant consequences, particularly in medical and biological contexts (189, 191). To prevent misdiagnosis and mistreatment that may lead to life-threatening conditions, the rationale and evidence for DL-provided conclusions must be clarified. Developing “explainable AI” (XAI) for accurate predictions with understandable assessment criteria should be further investigated as a future direction. More ambitiously, comprehending complex biological contexts, such as molecular mechanisms, genetic expression, and cellular microenvironments, is crucial for developing novel biomarkers, discovering disease pathogenesis, proposing new treatment strategies, and evaluating analytical approach performance (192, 193). All these advancements necessitate updating CNN architectures to not only make predictions based on data-driven DL approaches but also learn the biological mechanisms behind the data by integrating biological knowledge into the learning process (193, 194). A new concept called digital biopsy involves analyzing digital images and identifying characteristic features focusing on tumor heterogeneity rather than its contour, using computer power from multi-omics to aid in diagnosing or predicting various diseases (195). Investigating DL-based digital biopsy techniques will significantly contribute to assessing and predicting diseases non-invasively, making it a valuable tool in clinical settings. Digital biopsy holds considerable potential to become the “next-generation biopsy” for patients with low risk PCa, substantially benefiting healthcare.

## 5 Conclusion

DLAs have shown promising results in tumor identification and detection, lesion segmentation, PCa grading and scoring, as well as postoperative recurrence and prognostic outcome prediction, making them gain significant attention and play important roles in urology. However, the diagnostic accuracy of DL models still has room for improvement, and the amount of annotated sample data used is relatively limited. Therefore, optimization of models and algorithms, expansion of medical database resources, and combination of multi-omics data and comprehensive analysis of various morphological data will enhance the usefulness of DL for the diagnosis and treatment of urological diseases. Additionally, continued exploration in developing explainable AI will bring greater transparency and trustworthiness to DL. Undoubtedly, DL has shown a steep learning curve in the interpretation of prostate MRI (196), and its advent will benefit not only radiologists but also general physicians who lack systematic and specialized imaging interpretation training in terms of imaging evaluation of prostatic diseases. We believe that with advancements in technology and research, a significant leap in DLA development would occur, which would be beneficial in PCa diagnosis and treatment.

## Author contributions

MH performed the literature search regarding the available databases and drafted the manuscript. YC, XY and AK helped in consulting the relevant literature. RR, SW assisted in implementing images. OM, LZ and GY polished the manuscript. CC evaluated and reinfored the technical background. KH, ME contributed to editing the manuscript. All authors contributed to the article and approved the submitted version.
